# Insertion Multi-Parachute Suturing and Knotting (IMS-K) Technique for Aortic Surgery

**DOI:** 10.7759/cureus.53525

**Published:** 2024-02-03

**Authors:** Yoshinori Nakahara, Retsu Tateishi, Fumiya Haba, Syunya Ono, Takeyuki Kanemura

**Affiliations:** 1 Cardiovascular Surgery, IMS Katsushika Heart Center, Tokyo, JPN

**Keywords:** artificial vessel, parachute suturing, aortic dissection repair, aortic surgery, surgical anastomosis

## Abstract

Successful surgical interventions for aortic surgery, such as hemiarch repair and total arch replacement, pivot on the foundation of optimal anastomosis. We propose an alternative approach to anastomosis. The “insertion multi-parachute suturing and knotting” (IMS-K) technique entails the deployment of two parachute sutures, which can be effectively employed for both proximal and distal anastomoses. The first suture is applied loosely to the posterior half of the anastomosis and subsequently parachuted down, tightening the suture with a nerve hook. The second suture replicates the procedure in the anterior half of the anastomosis, loosely securing it in a similar manner and tightening it with a nerve hook at the end. As a result, the artificial graft is completely inserted into the aorta with a substantial grip. This technique simplifies the aortic anastomosis by ensuring procedural simplicity while minimizing bleeding risk, thus potentially advancing surgical outcomes.

## Introduction

The success of surgical procedures, such as hemiarch repair and total arch replacement, for aortic surgery relies heavily on distal or proximal anastomosis. Several anastomosis methods have been reported to achieve favorable outcomes (see Appendix), including the "turn-up" anastomosis technique for acute aortic dissection [1], the "tube-graft inversion" technique for constructing an open distal anastomosis during ascending aortic replacement [2], and the "double telescopic” anastomosis with the interrupted-suture technique [3]. However, there is currently no gold standard for anastomosis. In this report, we present a simple anastomosis technique that involves the full insertion of an artificial graft into the aorta using a multi-parachute technique.

## Technical report

**Figure 1:**
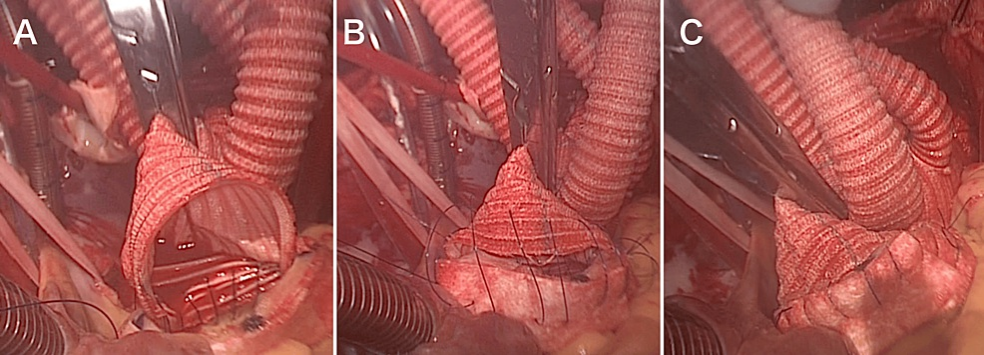
Proximal anastomosis in total arch replacement using the “insertion multi-parachute suturing and knotting” technique. (A) The first parachute stitch initiated at the 3 o'clock position traverses halfway around the circumference of the aorta counterclockwise and concludes at the 9 o'clock position. (B) The second parachute stitch threaded clockwise from the 3 o'clock to the 9 o'clock position, encompassing half the aorta's circumference. The threads were not tightened and were held loose. (C) The artificial graft is inserted into the aorta and the anastomosis is completed.

Video 1 demonstrates the “insertion multi-parachute suturing and knotting” (IMS-K) technique for proximal and distal anastomoses during total arch replacement that consists of two parachute sutures. The artificial graft and aorta are each marked to divide the anastomosis region into four equal segments. The artificial graft is positioned on the cephalic side of the aorta. First, an initial parachute stitch is executed with 3-0 or 4-0 polypropylene. This stitch was initiated at the 3 o'clock position, traversed halfway around the circumference of the aorta counterclockwise, and concluded at the 9 o'clock position (Figure 1A). This initial anastomosis encompasses half the circumference and typically involves nine stitches. All stitches were performed with the forehand, and the parachute stitch was securely tightened. At the 3 o'clock position, the first thread was pulled outwardly from the interior of the aorta, thereby allowing the artificial graft to be smoothly inserted into the aorta's interior. Second, a new thread was sutured at the 3 o'clock position and securely tied to the first thread to prevent loosening of the initial parachute thread. Subsequently, the second parachute stitch was threaded clockwise from the 3 o'clock to the 9 o'clock position, encompassing half of the circumference of the aorta with nine stitches (Figure 1B). All stitches were performed with the forehand. Finally, the artificial graft was manually inserted into the aorta, and the thread was meticulously tightened using a nerve hook. The thread at the 9 o'clock position was ligated with the first thread, culminating in the completion of the anastomosis (Figure 1C). 

**Video 1:** Surgical video of the “insertion multi-parachute suturing and knotting” technique in total arch replacement for thoracic aortic aneurysm.

## Discussion

**Figure 2:**
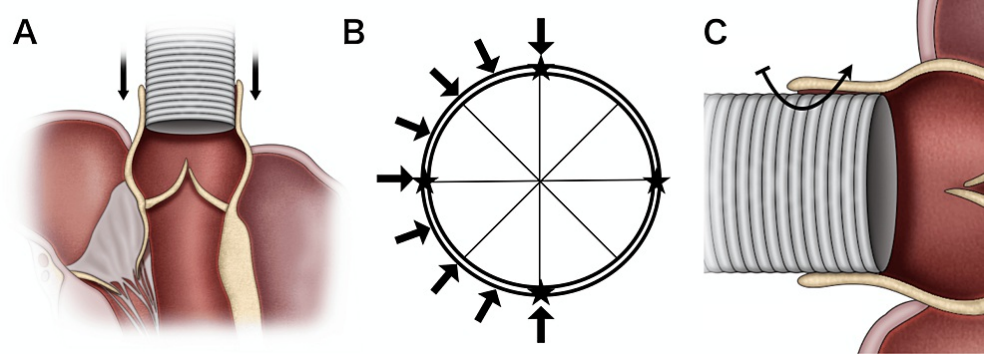
Schema of the insertion multi-parachute suturing and knotting (IMS-K) technique. (A) Complete insertion of the artificial graft into the aorta. (B) The anastomosis region is divided into four equal segments and three sutures are deployed across these four segments. The stars (*) indicate the point at which the artificial graft is divided into four parts. These are marked before anastomosis. The black arrows indicate the stitches’ points. (C) Effective shielding of the aortic wall from its interior by the artificial graft. The black arrow indicates the additional stitch for hemostasis.

The IMS-K technique offers four key advantages. First, it minimizes the risk of bleeding by inserting an artificial graft into the aorta with adequate bite, as illustrated in Figure 2A. Compared with a standard continuous suture, the bite can be sufficiently large, resulting in a substantial overlapping region between the artificial graft and the aorta. Several reported aortic anastomosis methods have similarly inserted an artificial graft into the aorta to achieve excellent hemostasis [1-4]. Their rationale was as follows: by completely inserting the artificial graft, the suture enhances the contact area between the artificial graft and the aortic wall, thereby reducing the likelihood of bleeding at the anastomotic site [4]. When antegrade blood flow occurs, the elevated systemic pressure propels the inner segment of the artificial graft against the outer aortic wall, promoting the sealing of the anastomosis and, consequently, diminishing bleeding [3]. This anastomosis obviates the need for a hemostatic agent, and there was no re-exploration for bleeding in more than 10 cases. Another anastomosis technique in which all artificial grafts are placed in the aorta before anastomosis and then withdrawn has also been reported [5]. Our anastomosis is more convenient for achieving the artificial graft insertion.

Second, a larger bite augments the pitch without inducing bleeding. We divide the anastomosis region into four equal segments, executing the anastomosis to deploy three sutures across these four segments, as shown in Figure 2B. Marking the four points on the artificial graft and aorta is crucial, which enables precise identification of the equally divided four. Assuming a 28-mm artificial graft, it is divided into 16 sections, each spanning approximately 5.5 mm. Although this pitch exceeds a standard continuous suture, it does not entail any bleeding concerns.

Third, in the event of additional suturing, the aortic wall is effectively protected from its interior by the artificial graft, rendering it safe for further needle placement (Figure 2C).

Finally, the IMS-K technique is technically uncomplicated and easily executed, with all stitches performed forehand in a two-dimensional manner; all anastomoses can be performed while viewing the inside of both the aorta and the artificial graft, allowing for precise needle handling. This is facilitated by the second parachute stitch, a novel aspect of the IMS-K technique. The second threads are deliberately not tightened and are held loosely while encompassing half of the circumference of the aorta. Furthermore, the assistant is relieved of the need to pull the thread each time, thereby preventing unexpected loosening of the thread. 

There are several notes on this technique. First, the force to pull the thread with a nerve hook must be appropriate to avoid damaging the thread. Moreover, in scenarios involving a larger artificial graft and a smaller aorta, employing a pliable graft ensures optimal insertion within the aorta. The soft artificial graft (J Graft®️, Japan Lifeline, Tokyo, Japan) is preferable in our method. Lastly, this technique may lead to more thread entanglement. However, awareness and careful thread organization can mitigate this concern.

## Conclusions

The IMS-K technique, involving the deployment of two parachute sutures, optimizes aortic anastomosis, rendering it technically simple and easy to perform. This method achieves meticulous needle handling and facilitates the complete insertion of the artificial graft, thereby potentially mitigating the risk of bleeding. Further studies and clinical validations are imperative to establish its efficacy and safety.

## Appendices

**Table 1:** Summary of techniques for aortic anastomosis

	Advantage	Disadvantage
Turn-up [1]	The cross-sectional exposure enables easy finding of bleeding points and additional stitches.	It requires double suturing, including a U-stay suture and continuous suture.
Tube-graft inversion [2]	It brings the entire circumference of the distal end of the inverted tube-graft virtually next to the entire circumference of the transected aorta, facilitating accurate suture placement. It decreases the number of stitches for anastomosis because the stitches pass through the graft and the aorta at once.	Antegrade cerebral perfusion cannot be used with this technique.
Double telescopic anastomosis [3]	Interrupted sutures decrease the cutting force caused by continuous running sutures on the aortic wall.	It requires double suturing, including a U-stay suture and continuous suture.
